# Eccrine poromatosis following hematopoietic stem cell transplantations

**DOI:** 10.1016/j.jdcr.2025.08.017

**Published:** 2025-08-29

**Authors:** Caitlin A. Kearney, Michael P. Lee, Ata S. Moshiri, Nicole Weiler

**Affiliations:** a The Ronald O. Perelman Department of Dermatology, New York University Grossman School of Medicine, New York, New York; b Division of Dermatology, Department of Medicine, New York University Grossman Long Island School of Medicine, Mineola, New York

**Keywords:** chemotherapy, eccrine poroma, eccrine poromatosis, hematopoietic stem cell transplantation, poroma, poromatosis

## Introduction

Eccrine poromas are benign adnexal neoplasms that arise from the acrosyringium or the intraepidermal portion of the eccrine sweat gland duct.^1^ Concurrent development of multiple poromas, termed poromatosis, is rare, with few cases reported in the literature, many of which involve patients with a history of hematologic malignancy and treatment with chemotherapy or radiation therapy.^2^^,^^3^ Here, we present a case of poromatosis in a patient with a history of aplastic anemia treated with allogenic hematopoietic stem cell transplantation (HSCT) and subsequent acute myeloid leukemia with myelodysplastic syndrome-related changes treated with induction chemotherapy and peripheral blood stem cell transplantation.

## Case report

A 66-year-old Hispanic man presented to his primary care physician with an asymptomatic rash on his back that had been present for several months. He had a history of aplastic anemia diagnosed 25 years prior to presentation, treated with allogenic HSCT. He subsequently experienced acute myeloid leukemia with myelodysplastic syndrome-related changes 7 years before presentation. He received cytosine arabinoside and daunorubicin induction chemotherapy followed by peripheral blood stem cell transplantation, achieving remission. The patient was immunosuppressed after transplantation with prednisone and tacrolimus. He experienced cutaneous graft-versus-host disease affecting 96% of his body surface area 6 years prior to presentation, which resolved after treatment with prednisone. Immunosuppressive agents were discontinued 5 years prior to presentation. Additional medical history included chemotherapy-induced peripheral neuropathy, stroke, and hypertension.

**Fig 1:**
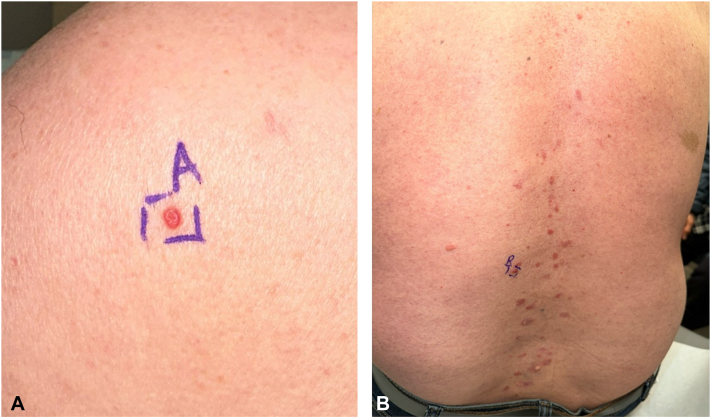
Clinical images of well-circumscribed, red and pink, dome-shaped papules located at the **(A)** left upper back and **(B)** left lower back.

**Fig 2:**
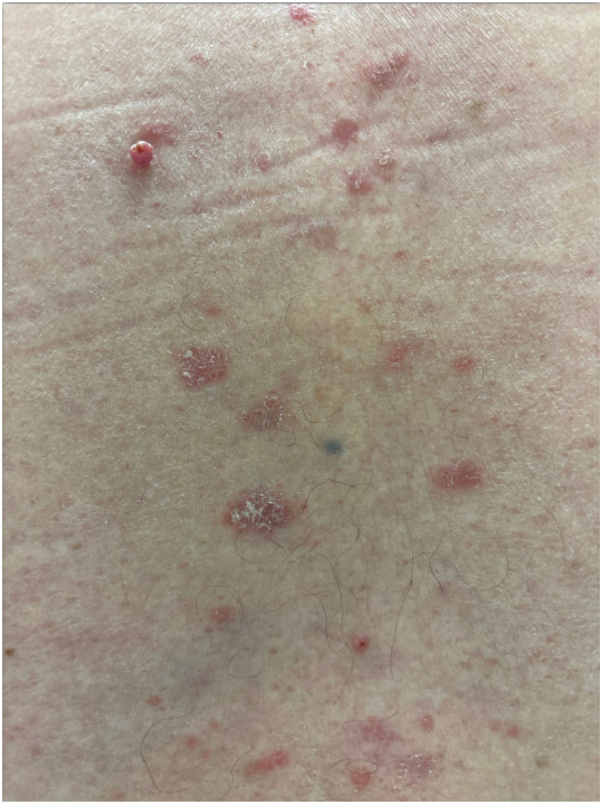
Clinical image of the lower back demonstrating a closer view of the dome-shaped pink papule and concomitant scaly erythematous plaques present at the lower back.

**Fig 3:**
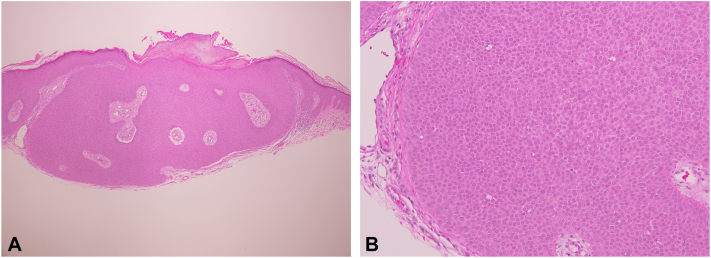
Histology from a shave biopsy of the left upper back lesion reveals a well-circumscribed epidermal proliferation of small, monomorphic cuboidal cells with compact eosinophilic cytoplasm and scattered small ductal lumina on higher power **(A, B)**.

**Fig 4:**
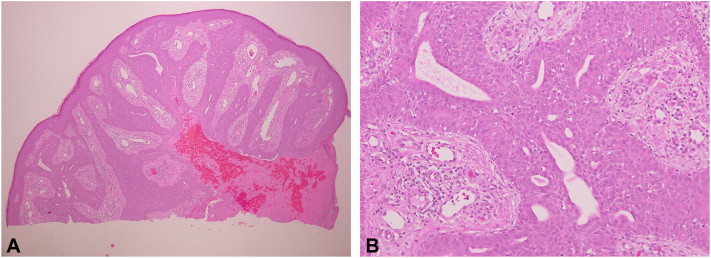
Histology from a shave biopsy of the left lower back lesion reveals a dome-shaped papule consisting of anastomosing epidermal strands of cuboidal cells, admixed with ductal lumina within a fibrovascular stroma with red blood cell extravasation **(A, B)**.

The patient’s primary care physician referred him to dermatology for further evaluation. Physical examination revealed 2 well-circumscribed, red, dome-shaped papules located on the left upper and lower back (Fig 1). The patient also exhibited scattered, well-circumscribed red plaques with scale across the lower back, clinically consistent with psoriasis, as well as a skin-colored papule consistent with an intradermal nevus, confirmed via dermatoscopic examination (Fig 2). Shave biopsies and histopathologic examination of the left upper and lower back lesions revealed well-circumscribed epidermal neoplasms composed of small monomorphous epithelial cells with scant cytoplasm and scattered small ducts lined by cuticular cells with eosinophilic cytoplasm, consistent with poromas (Figs 3 and 4). Given their benign nature, no immediate treatment was deemed necessary for the poromas, and ongoing surveillance of the lesions was recommended. The patient was prescribed 0.05% fluocinonide cream twice daily for psoriasis.

## Discussion

This case demonstrates poromatosis in a Hispanic man with a history of hematologic malignancy and 2 HSCTs. Poromas are relatively rare, comprising 10% of eccrine and apocrine sweat gland neoplasms, which represent 1% of primary cutaneous lesions.^1^ Patients with a history of chemotherapy or radiation therapy represent a distinct population at risk of eccrine poromatosis.^1^ The pathogenesis of poromatosis remains poorly understood, although it is hypothesized to involve secretion and concentration of toxic chemotherapy metabolites in eccrine glands, resulting in cellular remodeling and regeneration that predisposes to tumorigenesis.^4^ This mechanism parallels other eccrine toxic erythemas of chemotherapy, including neutrophilic eccrine hidradenitis and squamous syringometaplasia.^4^^,^^5^

The dual HSCT history in this case is notable, as cumulative chemotherapy exposure may increase the risk of eccrine toxicity. Of the poromatosis cases described in the literature, we identified 1 report of poromatosis in a patient who underwent more than 1 HSCT.^3^ Conditioning regimens prior to HSCT often include high-dose chemotherapy or full-body irradiation. There are multiple case reports documenting poromatosis in the setting of hematologic malignancies treated with HSCT, many of which arose multiple years after the conclusion of treatment.^2^^,^^3^^,^^6^ This case describes the development of poromatosis 7 years after the patient’s most recent peripheral blood stem cell transplantation, consistent with these prior reports and suggesting a delayed or lasting effect of chemotherapy on the eccrine gland.^6^

Although poromatosis is often observed in the context of chemotherapy, it remains rare among those who have received such treatment. Potential risk factors predisposing to an increased impact of chemotherapy on eccrine structures remain unknown. Many reports of poromatosis following chemotherapy arose in Japanese populations, leading authors to hypothesize the potential role of genetic or geographic predisposition.^2^^,^^4^ This case of poromatosis arose in a Hispanic man, contrasting with these reports and adding to the described demographics. We identified one other case of poromatosis in a Hispanic individual.^3^ Trauma and hyperhidrosis have also been proposed as potential risk factors.^6^

Rarely, eccrine poromas may transform into eccrine porocarcinoma, a malignant dermal duct neoplasm. Approximately 18% to 50% of eccrine porocarcinomas develop within a preexisting poroma.^1^ It remains unknown whether the risk of porocarcinoma in the setting of poromatosis varies from that of a solitary poroma. Clinical characteristics frequently observed alongside poromatosis, such as cancer history, cytotoxic chemotherapy exposure, or radiation exposure, could alter the potential for malignant transformation. Advanced age, chronic radiation exposure, and hematologic malignancies have been associated with porocarcinoma.^1^

There is 1 case report of porocarcinoma that presented concurrently with psoriasis in a Japanese man with no prior oncologic history.^7^ The presence of concomitant psoriasis alongside poromatosis represents an unusual clinical presentation. Psoriasis following allogenic HSCT is uncommon, as HSCT has been associated with remission or reduced flares of preexisting psoriasis rather than the development of de novo psoriasis posttransplant.^8^ Both poromatosis and psoriasis involve dysregulation of the yes-associated protein 1 (YAP1) pathway, although through distinct mechanisms. Recent case reports have identified YAP1:MAML2 gene fusions in poromatosis.^9^ YAP1 has also been found to be upregulated in psoriasis keratinocytes, promoting proliferation and inflammation.^10^ The coexistence of poromatosis and psoriasis in this patient may be coincidental, but raises the possibility of a shared predisposition via YAP1 pathway dysregulation in the context of prior immune-mediated skin injury.

The predisposition of poromatosis among patients with a history of hematologic malignancies treated with chemotherapy or HSCT highlights the importance of dermatologic surveillance in this population. These patients may be at increased risk of benign and malignant cutaneous lesions, including poromatosis, which may persist for years after the conclusion of treatment. This is a rare case of poromatosis in a patient with a history of hematologic malignancy and multiple HSCTs, which contributes to the growing body of literature suggesting a delayed effect of cumulative chemotherapy on eccrine structures, further increasing the risk of poromatosis.

## Conflicts of interest

None disclosed.
